# Risk of Cardiovascular Disease from Antiretroviral Therapy for HIV: A Systematic Review

**DOI:** 10.1371/journal.pone.0059551

**Published:** 2013-03-26

**Authors:** Clay Bavinger, Eran Bendavid, Katherine Niehaus, Richard A. Olshen, Ingram Olkin, Vandana Sundaram, Nicole Wein, Mark Holodniy, Nanjiang Hou, Douglas K. Owens, Manisha Desai

**Affiliations:** 1 Center for Primary Care and Outcomes Research, and Center for Health Policy, Stanford University, Stanford, California, United States of America; 2 Division of General Internal Medicine, Stanford University Medical Center, Stanford, California, United States of America; 3 Veterans Affairs Palo Alto Health Care System, Palo Alto, California, United States of America; 4 Division of Biostatistics, Stanford University Medical Center, Stanford, California, United States of America; 5 Department of Statistics, Stanford University, Stanford, California, United States of America; 6 Quantitative Sciences Unit, Department of Medicine, Stanford University Medical Center, Stanford, California, United States of America; Rush University, United States of America

## Abstract

**Background:**

Recent studies suggest certain antiretroviral therapy (ART) drugs are associated with increases in cardiovascular disease.

**Purpose:**

We performed a systematic review and meta-analysis to summarize the available evidence, with the goal of elucidating whether specific ART drugs are associated with an increased risk of myocardial infarction (MI).

**Data Sources:**

We searched Medline, Web of Science, the Cochrane Library, and abstract archives from the Conference on Retroviruses and Opportunistic Infections and International AIDS Society up to June 2011 to identify published articles and abstracts.

**Study Selection:**

Eligible studies were comparative and included MI, strokes, or other cardiovascular events as outcomes.

**Data Extraction:**

Eligibility screening, data extraction, and quality assessment were performed independently by two investigators.

**Data Synthesis:**

Random effects methods and Fisher’s combined probability test were used to summarize evidence.

**Findings:**

Twenty-seven studies met inclusion criteria, with 8 contributing to a formal meta-analysis. Findings based on two observational studies indicated an increase in risk of MI for patients recently exposed (usually defined as within last 6 months) to abacavir (RR 1.92, 95% CI 1.51–2.42) and protease inhibitors (PI) (RR 2.13, 95% CI 1.06–4.28). Our analysis also suggested an increased risk associated with each additional year of exposure to indinavir (RR 1.11, 95% CI 1.05–1.17) and lopinavir (RR 1.22, 95% CI 1.01–1.47). Our findings of increased cardiovascular risk from abacavir and PIs were in contrast to four published meta-analyses based on secondary analyses of randomized controlled trials, which found no increased risk from cardiovascular disease.

**Conclusion:**

Although observational studies implicated specific drugs, the evidence is mixed. Further, meta-analyses of randomized trials did not find increased risk from abacavir and PIs. Our findings that implicate specific ARTs in the observational setting provide sufficient evidence to warrant further investigation of this relationship in studies designed for that purpose.

## Introduction

Advances in HIV antiretroviral therapy (ART) have dramatically reduced mortality from HIV, such that a person receiving state-of-the-art ART may now expect to live 25 years and potentially longer [1]. Currently, about 50% of all patients with HIV die from causes considered unrelated to HIV [2]. Thus, management of HIV now involves the treatment of a chronic disease with the possibility of near normal life expectancy, but often with multiple comorbidities.

Recent studies suggest that some types of ART may be associated with increased risk of cardiovascular disease, a cause for concern given that people living with HIV may take ART for decades. The mechanisms causing an increased risk of cardiovascular disease are unclear, but according to a review by Grinspoon and Carr, “may relate to dyslipidemia, insulin resistance, diabetes mellitus, inflammation, impaired fibrinolysis, factors specific to antiretroviral medications, or combinations of these factors [3].” The authors further speculate that both HIV and ART might be associated with many of these risk factors [3].

Understanding the relationship between ART and cardiovascular risk is complex because the typical ART regimen contains at least three drugs from two drug classes, and many patients have had multiple regimens. Until recently, the three principal classes of ART have been: protease inhibitors (PIs), nucleoside reverse transcriptase inhibitors (NRTIs), and non-nucleoside reverse transcriptase inhibitors (NNRTIs). The evidence linking ART and cardiovascular disease has pointed specifically to PIs as a class, and specific agents (abacavir, didanosine) [4]–[7]. The evidence, however, has not been consistent. While some observational studies have found elevated risk with specific drugs or classes [4]–[7], another observational study has found contrasting evidence [8]. In addition, three recent meta-analyses of randomized trials evaluating abacavir, one of the implicated agents, did not find its exposure associated with an elevated risk of cardiovascular disease [9]–[11]. Our goal is to reconcile these inconsistencies. To that end, we performed a systematic review of studies that assess the risk of cardiovascular disease from ART. More specifically, we critically evaluated relevant studies to assess the strength of the evidence, to characterize the heterogeneity across studies, and when feasible to make use of information across studies in order to summarize statements regarding specific agents and classes.

## Methods

### Data Sources

We reviewed English-language articles on the association between antiretroviral drugs and cardiovascular outcomes published through 06/2011 in the Medline, Cochrane, and Web of Science databases, as well as abstracts from the two principal HIV-focused annual conferences: Conference on Retroviruses and Opportunistic Infections (CROI) and International AIDS Society (IAS). Our search terms included *myocardial infarction*, *stroke*, and *antiretroviral therapy*.

### Study Selection

**Figure 1:**
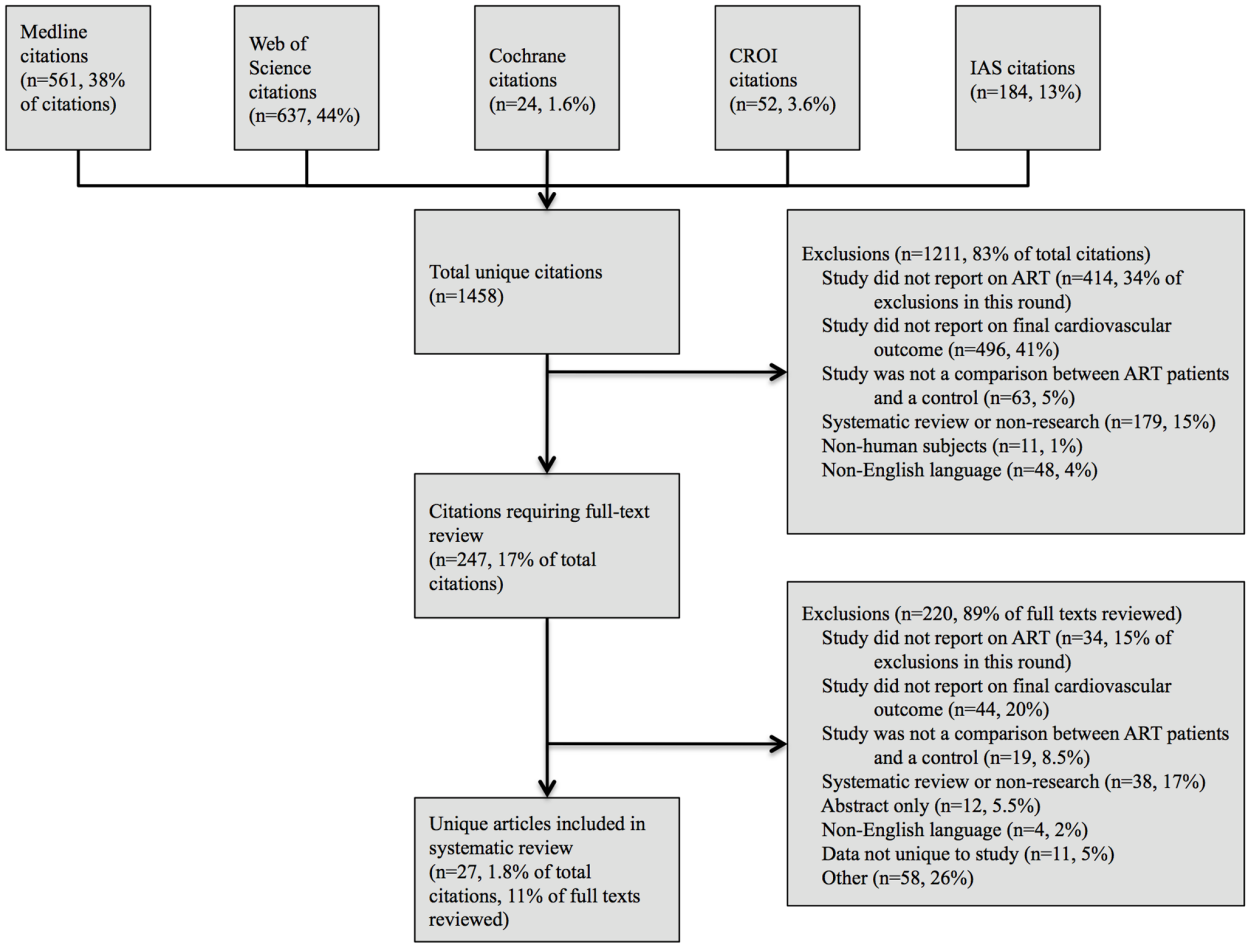
Study flow diagram.

We included comparative studies that described the association between antiretroviral drugs and cardiovascular events, including myocardial infarction (MI) and stroke. We included abstracts only when they presented unique data not already included in our analysis from published studies. Studies were excluded from our analysis if they were not comparative, if they only researched intermediate cardiovascular outcomes such as blood pressure, and if subjects were not humans infected with HIV (see Figure 1). Also, non-English language studies were excluded. Two investigators (from JB, KN, VS, or NH) independently reviewed titles, abstracts, and full articles to determine whether studies met inclusion criteria. Conflicting assessment between reviewers were resolved through discussion and review by the two assigned reviewers.

### Data Extraction

Two investigators (from JB, KN, VS, and NH) independently abstracted data on study design; eligibility and exclusion criteria; numbers of patients enrolled and lost to follow-up; method of treatment assessment; method of outcome assessment and results for each outcome.

### Quality Assessment

**Figure 2:**
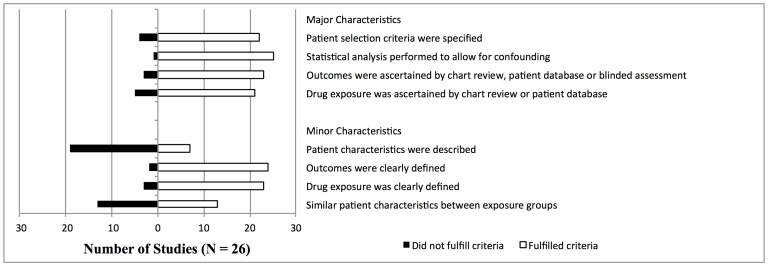
Quality of observational studies was judged according to 8 features of study design. The 4 major and 4 minor features are shown in this figure. Studies were rated as being of good, fair, or poor quality. Rating scheme is described in the Methods Section.

We assessed the quality of the study based on features of study design. For observational studies, we designed a rating scheme based on a methodological guide published by the Agency for Healthcare Research and Quality [12]. The most important (major) criteria were ascertainment of exposure, ascertainment of outcome, patient selection criteria, and use of adjusted analyses (see Figure 2). Additional criteria included similarity of patients between treatment and control groups, clear definition of exposure to drugs and outcomes, and adequate description of patient characteristics. We rated studies as good, fair, or poor. Only studies that clearly defined exposure to drugs, outcomes, and patient selection criteria, used medical chart review or chart linkage to gather patient exposure and outcome data, adjusted for common cardiovascular confounders, and fulfilled all quality criteria were rated as good. Ratings of fair were given to studies that fulfilled criteria for ascertainment of exposure and outcomes, patient selection, and used adjusted analyses, but that did not meet all of the additional quality criteria. Any study that failed one or more of the major criteria was rated as poor. All included studies stated approval by an appropriate research ethics committee, or were exempt from such approval because the study was a chart review using no identifying information.

We rated randomized clinical trials (RCTs) using the Jadad scale [13]. According to this system, RCT quality is based on whether the study was randomized, whether the study was double-blind, and whether there was a description of withdrawals from the study. For meta-analyses of RCTs, we used the AMSTAR rating system to rate each article numerically [14]. This system rates quality based on whether explicit inclusion criteria were developed before the search, whether a list of included and excluded articles was provided, whether quality of included articles was assessed, and whether proper statistics were used to combine evidence. Articles receiving a score of less than 5 were considered to be of poor methodological quality; articles with a score of between 5 to 8 were considered fair; and articles with a score of 9 or greater were considered to be good quality, as has been done previously [15].

### Data Synthesis and Analysis

We combined evidence from studies using two approaches. Our primary approach made use of random effects methods to combine point estimates of similar type [16] when a likelihood ratio test assessing heterogeneity was not rejected, implying the point estimates were not measuring inherently different quantities. Our secondary approach used Fisher’s method to combining p-values for summarizing evidence in cases where point estimates of different measures of risk were provided that could not be combined (for example, hazard ratios and odds ratios) [17], [18]. To ensure p-values across studies described associations in comparable directions we computed one-sided p-values for harm and one-sided p-values for protection and assessed significance of each at the 0.025 level. Finally, whereas our quantitative analyses addressed effects of regimens specifically on MI, we qualitatively compared the results from studies reporting on general cardiovascular events.

Our meta-analyses consisted of combining evidence for studies that addressed comparable questions. Because studies classified exposure differently and used different outcomes, we stratified analyses by two features: drug exposure (e.g., recent, usually defined as exposure within the last 6 months, or cumulative exposure measuring the number of years exposed to a drug or class) and the outcome of interest (MI, stroke, and any cardiovascular event). Even within a specific question (e.g., whether recent abacavir exposure affects MI relative to past abacavir exposure), study designs varied yielding variable types of estimates of risk including the relative risk (RR), the odds ratio (OR), or the hazard ratio (HR). Combining point estimates that measure different parameters is not recommended; more specifically, a combination of measures with different interpretations will not provide a summary statistic with a meaningful interpretation. Because MI is rare, however, the OR in this case may be viewed as a reasonable approximation of the RR, enabling us to formally combine evidence for studies that yield such estimates. All analyses were performed using the R statistical package (http://cran.r-project.org/) [19].

## Results

**Figure 3:**
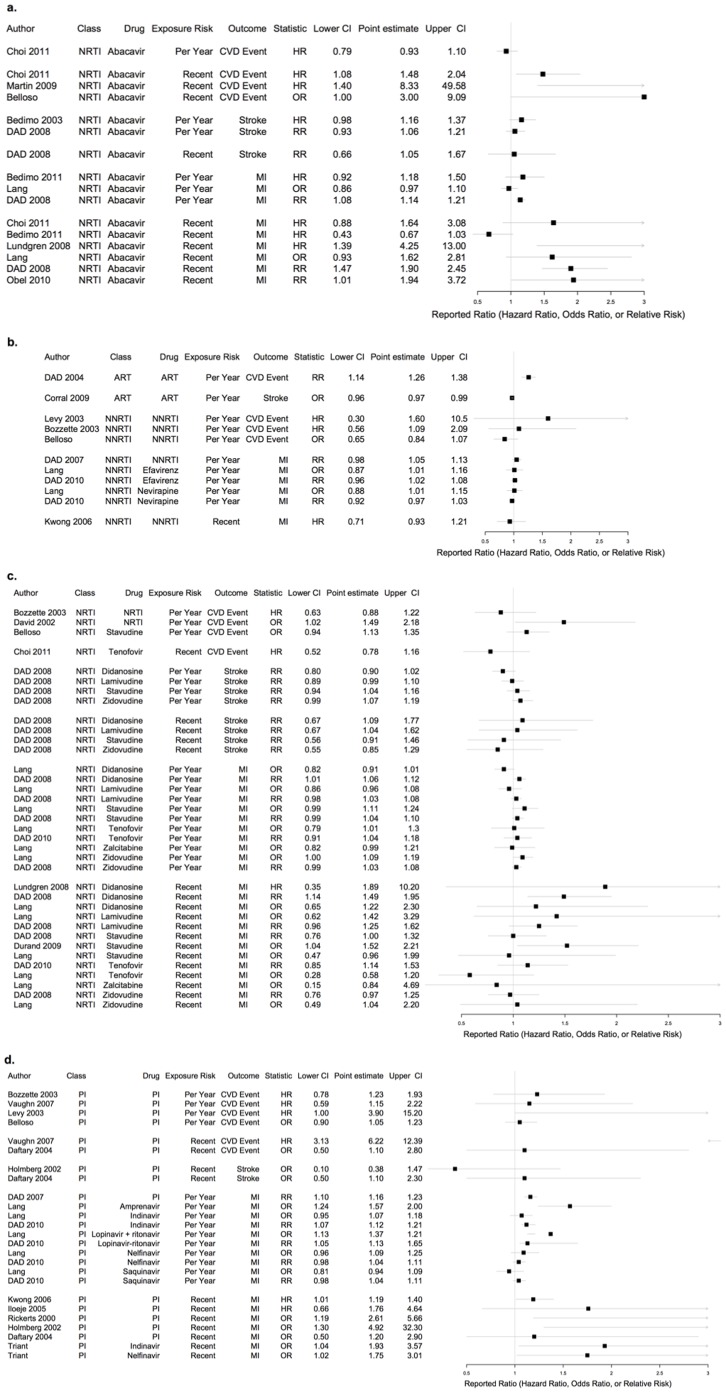
(a–d) Reported risk ratio and 95% confidence interval for each study group, organized by drug exposure, cardiovascular event, exposure definition, and risk ratio. Note that risk of recent exposure represents the effect of exposure to the agent within the past 6 months relative to non-exposure in the past 6 months, and risk per year represents the effect of one additional year of exposure to the agent.

We identified 1,458 articles; 27 met our inclusion criteria yielding 125 separate analyses (see Figure 1, Figure 3a–d, and Table 1). There was 1 RCT. All other studies were observational: 6 were case-control studies, and 20 were cohort studies. Of the observational studies, 5 studies were rated as good quality; 12 were rated as fair; and 9 were rated as poor (see Figure 2). The RCT was rated as good quality. We identified four meta-analyses of RCTs [9]–[11], [20]. Three of these [9]–[11] focused on abacavir, with significant overlap of studies analyzed. We therefore chose the meta-analysis that was most comprehensive [9], as well as the one meta-analysis of PI RCTs [20], and used the evidence from these as comparisons to our findings. Neither of these meta-analyses was rated as of good quality, because they did not provide a list of all included and excluded studies, did not provide an assessment of study quality, and did not assess likelihood of publication bias.

**Table 1:** Description of All Included Studies, ART, NNRTI, and NRTI.

Author	Year	Test Drug Class	Test Drug	Exposure Risk	Outcome	P-Value for Association	Point Estimate(95% CI)	Statistic	QualityRating
Durand	2009	ART	ART	Recent	Myocardial Infarction	Not significant	Not Reported	na	Poor
Levy	2003	ART	ART	Per Year	Cardiovascular Event	Not significant	2.2 (0.38–12.84)	HR	Fair
DAD	2004	ART	ART	Per Year	Cardiovascular Event	<.0001	1.26 (1.14–1.38)	RR	Fair
Corral	2009	ART	ART	Per Year	Stroke	0.002	0.97 (0.96–.99)	OR	Fair
Triant	2011	NNRTI	Efavirenz	Recent	Myocardial Infarction	Not significant	Not Reported	na	Poor
Lang	2010	NNRTI	Efavirenz	Per Year	Myocardial Infarction	0.94	1.01 (0.87–1.16)	OR	Fair
DAD	2010	NNRTI	Efavirenz	Per Year	Myocardial Infarction	Not significant	1.02 (0.96–1.08)	RR	Fair
Triant	2011	NNRTI	Nevirapine	Recent	Myocardial Infarction	Not significant	Not Reported	na	Poor
Lang	2010	NNRTI	Nevirapine	Per Year	Myocardial Infarction	0.95	1.01 (0.88–1.15)	OR	Fair
Kwong	2006	NNRTI	NNRTI	Recent	Myocardial Infarction	0.58	0.93 (0.71–1.21)	HR	Fair
Bozzette	2003	NNRTI	NNRTI	Per Year	Cardiovascular Event	0.97	1.04 (0.75–1.45)	HR	Good
Levy	2003	NNRTI	NNRTI	Per Year	Cardiovascular Event	Not significant	1.60 (0.19–13.29)	HR	Fair
David	2002	NNRTI	NNRTI	Per Year	Cardiovascular Event	0.09	Not Reported	na	Good
Belloso	2010	NNRTI	NNRTI	Per Year	Cardiovascular Event	0.157	0.84 (0.65–1.07)	OR	Poor
DAD	2007	NNRTI	NNRTI	Per Year	Myocardial Infarction	0.17	1.05 (0.98–1.13)	RR	Good
DAD	2010	NNRTI	Zalcitabine	Per Year	Myocardial Infarction	Not significant	Not Reported	na	Fair
Martin	2009	NRTI	Abacavir	Recent	Cardiovascular Event	0.048	8.33 (1.40–49.58)	HR	Good
Choi	2011	NRTI	Abacavir	Recent	Cardiovascular Event	0.015	1.48 (1.08–2.04)	HR	Good
Belloso	2010	NRTI	Abacavir	Recent	Cardiovascular Event	0.052	3.00 (1.00–9.09)	OR	Poor
Lundgren	2008	NRTI	Abacavir	Recent	Myocardial Infarction	Not reported	4.25 (1.39–13)	HR	Fair
Choi	2011	NRTI	Abacavir	Recent	Myocardial Infarction	Not reported	1.64 (0.88–3.08)	HR	Good
Bedimo	2011	NRTI	Abacavir	Recent	Myocardial Infarction	0.07	0.67 (0.43–1.03)	HR	Fair
Triant	2011	NRTI	Abacavir	Recent	Myocardial Infarction	Not significant	Not Reported	na	Poor
Durand	2009	NRTI	Abacavir	Recent	Myocardial Infarction	Not significant	Not Reported	na	Poor
Lang	2010	NRTI	Abacavir	Recent	Myocardial Infarction	0.09	1.62 (0.93–2.81)	OR	Fair
DAD	2008	NRTI	Abacavir	Recent	Myocardial Infarction	0.0001	1.9 (1.47–2.45)	RR	Fair
Obel	2010	NRTI	Abacavir	Recent	Myocardial Infarction	Not reported	1.94 (1.01–3.72)	RR	Poor
DAD	2008	NRTI	Abacavir	Recent	Stroke	0.84	1.05 (0.66–1.67)	RR	Fair
Choi	2011	NRTI	Abacavir	Per Year	Cardiovascular Event	Not reported	0.93 (0.79–1.10)	HR	Good
Bedimo	2011	NRTI	Abacavir	Per Year	Myocardial Infarction	0.191	1.18 (0.92–1.50)	HR	Fair
Lang	2010	NRTI	Abacavir	Per Year	Myocardial Infarction	0.64	0.97 (0.86–1.1)	OR	Fair
DAD	2008	NRTI	Abacavir	Per Year	Myocardial Infarction	0.0001	1.14 (1.08–1.21)	RR	Fair
Bedimo	2003	NRTI	Abacavir	Per Year	Stroke	0.096	1.16 (0.98–1.37)	HR	Poor
DAD	2008	NRTI	Abacavir	Per Year	Stroke	0.4	1.06 (0.93–1.21)	RR	Fair
Durand	2009	NRTI	Abacavir	Ever	Myocardial Infarction	Significant	1.74 (1.18–2.56)	OR	Poor
Lundgren	2008	NRTI	Didanosine	Recent	Myocardial Infarction	Not reported	1.89 (0.35–10.20)	HR	Fair
Triant	2011	NRTI	Didanosine	Recent	Myocardial Infarction	Not significant	Not Reported	na	Poor
Durand	2009	NRTI	Didanosine	Recent	Myocardial Infarction	Not significant	Not Reported	na	Poor
Lang	2010	NRTI	Didanosine	Recent	Myocardial Infarction	0.54	1.22 (0.65–2.30)	OR	Fair
DAD	2008	NRTI	Didanosine	Recent	Myocardial Infarction	0.003	1.49 (1.14–1.95)	RR	Fair
DAD	2008	NRTI	Didanosine	Recent	Stroke	0.74	1.09 (0.67–1.77)	RR	Fair
Lang	2010	NRTI	Didanosine	Per Year	Myocardial Infarction	0.06	0.91 (0.82–1.01)	OR	Fair
DAD	2008	NRTI	Didanosine	Per Year	Myocardial Infarction	0.03	1.06 (1.01–1.12)	RR	Fair
DAD	2008	NRTI	Didanosine	Per Year	Stroke	0.09	0.9 (0.8–1.02)	RR	Fair
Durand	2009	NRTI	Didanosine	Ever	Myocardial Infarction	Significant	1.60 (1.06–2.43)	OR	Poor
Triant	2011	NRTI	Emtricitabine	Recent	Myocardial Infarction	Not significant	Not Reported	na	Poor
Durand	2009	NRTI	Emtricitabine	Recent	Myocardial Infarction	Not significant	Not Reported	na	Poor
Durand	2009	NRTI	Emtricitabine	Ever	Myocardial Infarction	Not significant	Not Reported	na	Poor
Triant	2011	NRTI	Lamivudine	Recent	Myocardial Infarction	Not significant	Not Reported	na	Poor
Durand	2009	NRTI	Lamivudine	Recent	Myocardial Infarction	Not significant	Not Reported	na	Poor
Lang	2010	NRTI	Lamivudine	Recent	Myocardial Infarction	0.41	1.42 (0.62–3.29)	OR	Fair
DAD	2008	NRTI	Lamivudine	Recent	Myocardial Infarction	0.1	1.25 (0.96–1.62)	RR	Fair
DAD	2008	NRTI	Lamivudine	Recent	Stroke	0.86	1.04 (0.67–1.62)	RR	Fair
Lang	2010	NRTI	Lamivudine	Per Year	Myocardial Infarction	0.52	0.96 (0.86–1.08)	OR	Fair
DAD	2008	NRTI	Lamivudine	Per Year	Myocardial Infarction	0.28	1.03 (0.98–1.08)	RR	Fair
DAD	2008	NRTI	Lamivudine	Per Year	Stroke	0.89	.99 (0.89–1.1)	RR	Fair
Durand	2009	NRTI	Lamivudine	Ever	Myocardial Infarction	Not significant	Not Reported	na	Poor
Bozzette	2003	NRTI	NRTI	Per Year	Cardiovascular Event	0.72	0.94 (0.80–1.11)	HR	Good
David	2002	NRTI	NRTI	Per Year	Cardiovascular Event	0.04	1.49 (1.02–2.18)	OR	Good
Triant	2011	NRTI	Stavudine	Recent	Myocardial Infarction	Not significant	Not Reported	na	Poor
Durand	2009	NRTI	Stavudine	Recent	Myocardial Infarction	Significant	1.52 (1.04–2.21)	OR	Poor
Lang	2010	NRTI	Stavudine	Recent	Myocardial Infarction	0.92	0.96 (0.47–1.99)	OR	Fair
DAD	2008	NRTI	Stavudine	Recent	Myocardial Infarction	0.98	1.00 (0.76–1.32)	RR	Fair
DAD	2008	NRTI	Stavudine	Recent	Stroke	0.69	0.91 (0.56–1.46)	RR	Fair
Belloso	2010	NRTI	Stavudine	Per Year	Cardiovascular Event	0.187	1.13 (0.94–1.35)	OR	Poor
Lang	2010	NRTI	Stavudine	Per Year	Myocardial Infarction	0.07	1.11 (0.99–1.24)	OR	Fair
DAD	2008	NRTI	Stavudine	Per Year	Myocardial Infarction	0.11	1.04 (0.99–1.1)	RR	Fair
DAD	2008	NRTI	Stavudine	Per Year	Stroke	0.47	1.04 (0.94–1.16)	RR	Fair
Durand	2009	NRTI	Stavudine	Ever	Myocardial Infarction	Significant	1.50 (1.07–2.12)	OR	Poor
Choi	2011	NRTI	Tenofovir	Recent	Cardiovascular Event	0.22	0.78 (0.52–1.16)	HR	Good
Triant	2011	NRTI	Tenofovir	Recent	Myocardial Infarction	Not significant	Not Reported	na	Poor
Durand	2009	NRTI	Tenofovir	Recent	Myocardial Infarction	Not significant	Not Reported	na	Poor
Lang	2010	NRTI	Tenofovir	Recent	Myocardial Infarction	0.14	0.58 (0.28–1.20)	OR	Fair
DAD	2010	NRTI	Tenofovir	Recent	Myocardial Infarction	Not significant	1.14 (0.85–1.53)	RR	Fair
Lang	2010	NRTI	Tenofovir	Per Year	Myocardial Infarction	0.95	1.01 (0.79–1.3)	OR	Fair
DAD	2010	NRTI	Tenofovir	Per Year	Myocardial Infarction	Not significant	1.04 (0.91–1.18)	RR	Fair
Durand	2009	NRTI	Tenofovir	Ever	Myocardial Infarction	Not significant	Not Reported	na	Poor
Durand	2009	NRTI	Zalcitabine	Recent	Myocardial Infarction	Not significant	Not Reported	na	Poor
Lang	2010	NRTI	Zalcitabine	Recent	Myocardial Infarction	0.84	0.84 (0.15–4.69)	OR	Fair
Lang	2010	NRTI	Zalcitabine	Per Year	Myocardial Infarction	0.95	0.99 (0.82–1.21)	OR	Fair
Durand	2009	NRTI	Zalcitabine	Ever	Myocardial Infarction	Not significant	Not Reported	na	Poor
Triant	2011	NRTI	Zidovudine	Recent	Myocardial Infarction	Not significant	Not Reported	na	Poor
Durand	2009	NRTI	Zidovudine	Recent	Myocardial Infarction	Not significant	Not Reported	na	Poor
Lang	2010	NRTI	Zidovudine	Recent	Myocardial Infarction	0.93	1.04 (0.49–2.20)	OR	Fair
DAD	2008	NRTI	Zidovudine	Recent	Myocardial Infarction	0.82	0.97 (0.76–1.25)	RR	Fair
DAD	2008	NRTI	Zidovudine	Recent	Stroke	0.44	0.85 (0.55–1.29)	RR	Fair
Lang	2010	NRTI	Zidovudine	Per Year	Myocardial Infarction	0.05	1.09 (1.00–1.19)	OR	Fair
DAD	2008	NRTI	Zidovudine	Per Year	Myocardial Infarction	0.14	1.03 (0.99–1.08)	RR	Fair
DAD	2008	NRTI	Zidovudine	Per Year	Stroke	0.1	1.07 (0.99–1.19)	RR	Fair
Durand	2009	NRTI	Zidovudine	Ever	Myocardial Infarction	Not significant	Not Reported	na	Poor
Lang	2010	PI	Amprenavir	Per Year	Myocardial Infarction	0.001	1.57 (1.24–2.00)	OR	Fair
Triant	2011	PI	Atazanavir	Recent	Myocardial Infarction	Not significant	Not Reported	na	Poor
Lang	2010	PI	Indinavir	Per Year	Myocardial Infarction	0.29	1.07 (0.95–1.21)	OR	Fair
DAD	2010	PI	Indinavir	Per Year	Myocardial Infarction	Not significant	1.12 (1.07–1.18)	RR	Fair
Triant	2011	PI	Indinavir	Recent	Myocardial Infarction	Not significant	1.93 (1.04–3.57)	OR	Poor
Triant	2011	PI	Lopinavir	Recent	Myocardial Infarction	Not significant	Not Reported	na	Poor
Lang	2010	PI	Lopinavir	Per Year	Myocardial Infarction	0.002	1.37 (1.13–1.65)	OR	Fair
DAD	2010	PI	Lopinavir	Per Year	Myocardial Infarction	Not significant	1.13 (1.05–1.21)	RR	Fair
Triant	2011	PI	Nelfinavir	Recent	Myocardial Infarction	Not significant	1.75 (1.02–3.01)	OR	Poor
Lang	2010	PI	Nelfinavir	Per Year	Myocardial Infarction	0.2	1.09 (0.96–1.25)	OR	Fair
DAD	2010	PI	Nelfinavir	Per Year	Myocardial Infarction	Not significant	1.04 (0.98–1.11)	RR	Fair
DAD	2010	PI	Nevirapine	Per Year	Myocardial Infarction	Not significant	0.97 (0.92–1.03)	RR	Fair
Vaughn	2007	PI	PI	Recent	Cardiovascular Event	<.001	6.22 (3.13–12.39)	HR	Good
Daftary	2004	PI	PI	Recent	Congestive Heart Failure	Not significant	1.1 (0.5–2.8)	OR	Poor
Iloeje	2005	PI	PI	Recent	Myocardial Infarction	Not reported	1.76 (0.66–4.64)	HR	Poor
Kwong	2006	PI	PI	Recent	Myocardial Infarction	0.04	1.19 (1.01–1.40)	HR	Fair
Quiros-Roldan	2005	PI	PI	Recent	Myocardial Infarction	Not significant	Not Reported	na	Fair
Daftary	2004	PI	PI	Recent	Myocardial Infarction	Not significant	1.2 (0.5–2.9)	OR	Poor
Holmberg	2002	PI	PI	Recent	Myocardial Infarction	0.04	4.92 (1.3–32.3)	OR	Poor
Rickerts	2000	PI	PI	Recent	Myocardial Infarction	0.01	2.61 (1.19–5.66)	OR	Poor
Barbaro	2003	PI	PI	Recent	Myocardial Infarction	<.001	Not Reported	RR	Fair
Iloeje	2005	PI	PI	Recent	Stroke	Not reported	Not Reported	na	Poor
Daftary	2004	PI	PI	Recent	Stroke	Not significant	1.1 (0.5–2.3)	OR	Poor
Holmberg	2002	PI	PI	Recent	Stroke	0.206	0.38 (0.1–1.47)	OR	Poor
Bozzette	2003	PI	PI	Per Year	Cardiovascular Event	0.57	1.11 (0.88–1.39)	HR	Good
Levy	2003	PI	PI	Per Year	Cardiovascular Event	Not significant	3.9 (0.77–19.84)	HR	Fair
Vaughn	2007	PI	PI	Per Year	Cardiovascular Event	0.682	1.15 (0.59–2.22)	HR	Good
David	2002	PI	PI	Per Year	Cardiovascular Event	0.46	Not Reported	na	Good
Belloso	2010	PI	PI	Per Year	Cardiovascular Event	0.512	1.05 (0.90–1.23)	OR	Poor
DAD	2007	PI	PI	Per Year	Myocardial Infarction	<.001	1.16 (1.10–1.23)	RR	Good
Klein	2002	PI	PI	Ever	Myocardial Infarction	Not significant	Not Reported	RR	Fair
Triant	2011	PI	Ritonavir	Recent	Myocardial Infarction	Not significant	Not Reported	na	Poor
Triant	2011	PI	Saquinavir	Recent	Myocardial Infarction	Not significant	Not Reported	na	Poor
Lang	2010	PI	Saquinavir	Per Year	Myocardial Infarction	0.39	0.94 (0.81–1.09)	OR	Fair
DAD	2010	PI	Saquinavir	Per Year	Myocardial Infarction	Not significant	1.04 (0.98–1.11)	RR	Fair

Description of all included studies. All included studies and exposure groups are listed. Author, test drug, exposure risk, cardiovascular outcome, result, and quality rating are provided. Note that risk of recent exposure represents the effect of exposure to the agent within the past 6 months relative to non-exposure in the past 6 months, and risk per year represents the effect of one additional year of exposure to the agent.

We were able to combine results from 8 observational studies [4], [6], [8], [21]–[25] that described associations using odds ratios in a formal meta-analysis. The remaining observational studies reported associations using hazard ratios [26]–[30], did not report MI-specific cardiovascular outcomes [29], [31]–[38], were the only study reporting on a specific drug exposure [7], [39], or did not provide a quantified measure of their findings (only whether the findings were significant or not) [40]–[42]. As some studies investigated multiple drugs, they may have contributed to separate analyses of more than one drug class in our meta-analysis (see Tables 2, 3). Table 2 shows the exposures for which we were able to provide summary estimates of effect size, and Table 3 indicates the exposures for which we were able to combine p-values using Fisher’s method.

**Table 2:** Summary of Systematic Review.

Exposures:	CumulativeNNRTI		CumulativeNRTI						RecentNRTI						CumulativePI				Recent PI
Study	Efa	Nev	Zid	Sta	Lam	Ten	Aba	Did	Zid	Sta	Lam	Ten	Aba*	Did	Nel	Ind*	Lop*	Saq	General*
DAD 2008			X	X	X		>	>	X	X	X		X	X					
DAD 2010	X	X				X						X			X	X	X	X	
Lang	X	X	X	X	X	X	<	<	+	+	+	+	+	+	X	X	X	X	
Durand										X									
Obel													X						
Daftary																			X
Holmberg																			X
Rickerts																			X
Disagree							Yes	Yes											

Exposures investigated by lead author of study. An “X” indicates whether the agent was evaluated by a particular study. The last row indicates whether there was sufficient evidence to demonstrate the studies estimated heterogeneous effects. Drugs with an asterisk (*) next to their names identify those implicated by the meta-analysis. Plus signs (+) indicate exclusion from analysis due to incompatible definition of exposure and/or reference group. “Greater Than” symbols (>) and “Less Than” symbols (<) indicate the test for heterogeneity was rejected and indicate whether the point estimate indicated harm (>) or protection (<), although this effect was not necessarily significant. Abbreviations: Efa, Efavirenz; Nev, Nevarapine; Zid, Zidovudine; Sta, Stavudine; Lam, Lamivudine; Ten, Tenofovir; Aba, Abacavir; Did, Didanosine; Nel, Nelfinavir; Ind, Indinavir; Lop, Lopinavir; Saq, Saquinavir.

**Table 3:** Supplemental Summary of Association by P-Value.

Exposures:	Recent NRTI						Cumulative NRTI		Recent PI
Study	Aba*	Did*	Lam	Sta	Ten	Zid	Aba*	Did	General*
Barbaro									X
Bedimo							X		
Choi	X								
DAD 2008	X	X	X	X		X	X	X	
DAD 2010					X				
Daftary									X
Durand				X					
Holmberg									X
Iloeje									X
Kwong									X
Lang	X	X	X	X	X	X	X	X	
Lundgren	X	X							
Obel	X								
Rickerts									X

Studies included in a supplemental analysis that compiled evidence not included in the summary of point estimates, either because the point estimates were not presented as odds ratios or relative risks, or because the exposure definitions between studies differed too significantly. Here, more studies have been included. This analysis was only performed on exposure groups which have additional evidence included compared to the point estimate analysis. An “X” indicates whether the exposure was evaluated by a particular study. Summary p-values describing the evidence of association based on Fisher’s method are presented in Table 4. Exposure groups with an asterisk (*) next to their names were implicated by this analysis. Abbreviations: Aba, Abacavir; Did, Didanosine; Lam, Lamivudine; Sta, Stavudine; Ten, Tenofovir; Zid, Zidovudine.

### Yearly Exposure to NRTIs

Two studies reported cardiovascular risk assessed based on yearly exposure to NRTIs [4], [8]. We were unable to combine evidence on yearly use of abacavir due to heterogeneity of the results from the two reporting studies. Specifically, while Lang et al. [8] showed no association between yearly use of abacavir and risk of MI (OR 0.97, 95% CI: 0.86, 1.1), DAD reported a relative risk of 1.14 (95% CI: 1.08, 1.21) [4].

These two studies showed similar heterogeneity when assessing risk from yearly exposure to didanosine; Lang et al. reported no association and the DAD reported an increased risk (0.91, 95% CI: 0.82, 1.01 and 1.06, 95% CI: 1.01, 1.12).

No significant findings were observed in our meta-analysis for cumulative exposure to the other NRTI agents investigated (lamivudine, stavudine, tenofovir and zidovudine exposure).

### Recent Exposure to NRTIs

**Figure 4:**
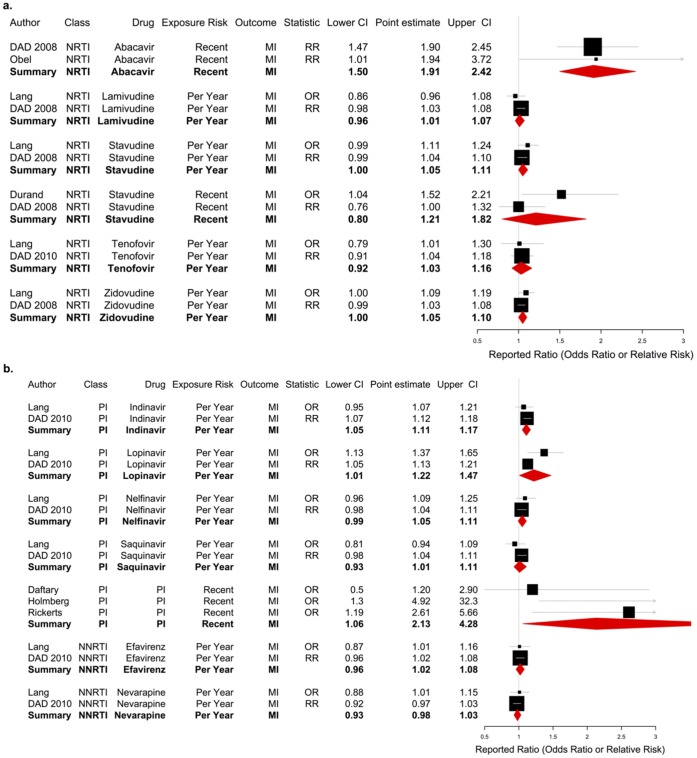
(a, b) Reported risk ratio and 95% confidence interval for exposure groups with sufficient evidence to summarize in meta-analysis. Results of meta-analysis are shown in bottom row of each exposure group, denoted pictorially by the red diamond. Each study is given a weight based on its number of subjects and length of follow-up, denoted pictorially by size of its box in the plot. Note that risk of recent exposure represents the effect of exposure to the agent within the past 6 months relative to non-exposure in the past 6 months, and risk per year represents the effect of one additional year of exposure to the agent.

Our pooled analysis of 2 studies [4], [21] demonstrated an association between recent exposure (usually defined as within last 6 months) of abacavir and risk of MI, with a summary RR of 1.91 (95% CI: 1.50, 2.42, see Figure 4a). In addition, combining p-values across all studies that evaluate comparable definitions of recent exposure to abacavir with comparable reference groups (See Table 4) using Fisher’s method suggested a harmful association between recent abacavir use and MI (p<0.001).

**Table 4:** Results of Fisher’s P-Value Test.

Exposure Group	Study	Point Estimate (95% CI)	Statistic	P-Value Association	P-Value ofHarm	P-Value of Protection
Abacavir, Risk Per Year						
	Bedimo	1.18 (0.92–1.50)	HR	0.191	0.0922	0.908
	Lang	0.97 (0.86–1.10)	OR	0.64	0.6862	0.3138
	DAD 2008	1.14 (1.08–1.21)	RR	0.0001	0.000003106	1
	Summary			0.000932618	2.66622E−05	0.8672322
						
Abacavir, Risk of Recent Exposure						
	Choi	1.64 (0.88–3.08)	HR	0.1216353	0.06082	0.9391
	Lundgren	4.25 (1.39–13.00)	HR	0.01117942	0.00559	0.9944
	Lang	1.62(0.93–2.81)	OR	0.09	0.04361	0.9563
	DAD 2008	1.9 (1.47–2.45)	RR	0.0001	4.207E−07	1
	Obel	1.94 (1.01–3.72)	RR	0.04632	0.02316	0.9768
	Summary			5.80959E−06	3.87002E−08	0.9999996
						
Didanosine, Risk Per Year						
	Lang	0.91 (0.82–1.01)	OR	0.06	0.962	0.03803
	DAD 2008	1.06 (1.01–1.12)	RR	0.03	0.01357	0.9864
	Summary			0.01317594	0.06969235	0.1605729
						
Didanosine, Risk of Recent Exposure						
	Lundgren	1.89 (0.35–10.20)	HR	0.4593097	0.2297	0.7703
	Lang	1.22 (0.65–2.30)	OR	0.54	0.2687	0.7313
	DAD 2008	1.49 (1.14–1.95)	RR	0.003	0.001795	0.9982
	Summary			0.02539555	0.005714974	0.9792164
						
Lamivudine, Risk of Recent Exposure						
	Lang	1.42 (0.62–3.29)	OR	0.41	0.2051	0.7949
	DAD 2008	1.25 (.96–1.62)	RR	0.1	0.04729	0.9527
	Summary			0.1719615	0.0546618	0.9678266
						
Stavudine, Risk of Recent Exposure						
	Durand	1.52 (1.04–2.21)	OR	0.02944	0.01472	0.985
	Lang	0.96 (0.47–1.99)	OR	0.92	0.5441	0.4559
	DAD 2008	1.00 (0.76–1.32)	RR	0.98	0.5	0.5
	Summary			0.2976478	0.08712864	0.810416
						
Tenofovir, Risk of Recent Exposure						
	Lang	.058 (0.28–1.20)	OR	0.14	0.9289	0.0711
	DAD 2010	1.04 (0.91–1.18)	RR	0.5540343	0.277	0.723
	Summary			0.2758579	0.6065952	0.203977
						
Zidovudine, Risk of Recent Exposure						
	Lang	1.04 (0.49–2.20)	OR	0.93	0.4592	0.5408
	DAD 2008	0.97 (0.76–1.25)	RR	0.82	0.5948	0.4052
	Summary			0.969281	0.627603	0.5517924
						
Protease Inhibitor, Risk of Recent Exposure						
	Iloeje	1.76 (0.66–4.64)	HR	0.2558345	0.1279	0.872
	Kwong	1.19 (1.01–1.40)	HR	0.04	0.01838	0.98162
	Barbaro	Not Reported	RR	0.001	Cannot Calculate	Cannot Calculate
	Holmberg	4.92 (1.30–32.3)	OR	0.04	0.02594	0.974
	Rickerts	2.61 (1.19–5.66)	OR	0.01	0.007944	0.992
	Daftary	1.20 (0.50 –2.90)	OR	0.6843204	0.3422	0.6577
	Summary			9.08759E−05	0.000538497	0.9995785

Results of Fisher’s P-value test, organized by drug and exposure definition. P-values are listed as overall p-value of association, and split into one-tailed p-values of harm and protection. Results are listed in bottom row of each group, denoted in bold text. Results are listed for any study group for which the Fisher’s test included more studies than the main meta-analysis. Note that risk of recent exposure represents the effect of exposure to the agent within the past 6 months relative to non-exposure in the past 6 months, and risk per year represents the effect of one additional year of exposure to the agent.

Both DAD [4] and Lang et al. [8] assessed the association between recent exposure to didanosine and MI, but because a test of heterogeneity indicated the parameters were incompatible, we did not combine the evidence. However, using Fisher’s method of combining p-values, we were able to include these as well as the results of Lundgren [28] (see Table 4). The results indicated a harmful association (p = 0.001). One additional study found no association between didanosine use and MI, but could not contribute to quantitative analyses, as they did not report numerical results [39].

No significant findings were observed in our meta-analysis for recent exposure to the other NRTI agent investigated (stavudine).

### Yearly Exposure to PIs

Cumulative exposure to individual PIs was investigated by the DAD and by Lang et al. [6], [8] (see Table 2). Combining evidence from these two studies demonstrated significantly increased risks of MI with cumulative indinavir use (1.11, 95% CI: 1.05, 1.17) and cumulative use of lopinavir with or without ritonavir (1.22, 95% CI: 1.01, 1.47). Lang et al. also found an increased risk associated with amprenavir, although no other studies identified such an association. Neither nelfinavir nor saquinavir were found to be significantly harmful in any study. Only one study by Friis-Moller et al. examined the effect of cumulative exposure to PIs as an entire class on MI, where a significantly increased risk of MI was observed [7].

### Recent Exposure to PIs

Recent PI exposure was examined by nine studies, with five finding significantly increased cardiovascular risk (see Figure 3d). Summarizing the three studies that reported odds ratios for MI [23]–[25] yielded an OR of 2.13 (95% CI: 1.06, 4.28) (see Figure 4b). Using Fisher’s method of summarizing p-values for the six studies reporting on recent PI use and MI [23]–[27], [41] we found an overall significant risk for MI associated with recent exposure to PIs as a class (summary p-value = 0.003). One additional study investigated PI drugs individually, finding a 75% and 93% increased risk of MI (95% CIs: 1.02, 3.01 and 1.04, 3.57) for nelfinavir and indinavir, respectively [39].

## Discussion

Our analysis combined evidence across studies investigating the association between cumulative and recent exposure to specific ART drugs as well as to classes of ART drugs and the risk of MI. Our findings implicated recent exposure to abacavir, recent exposure to PIs in general, and cumulative exposure to PIs indinavir and lopinavir. There are several issues, however, that need to be considered when interpreting our findings.

### Exposure to Abacavir

While our meta-analysis suggested an association between recent abacavir use and risk of MI, we note there were inconsistencies across studies in both findings and study quality. Six studies reported the association between recent abacavir use and risk of MI: three reported significant increases in risk [4], [21], [28]. One of these six studies was of good quality [30], four were of fair quality [4], [8], [28], [43], and one other was of poor quality; its patients were not similar across control and treatment groups [21]. Thus, although the observational studies point towards an increase in MI risk (Figure 3a), the evidence is not fully consistent. In contrast, three meta-analyses of randomized clinical trials reported no evidence of an association between abacavir use and MI [9], [10], [11]. Of the three studies, the meta-analysis by Ding et al. included a greater number of studies, including those used in the other two analyses, so we chose to compare our results to those from the study by Ding et al. [9], [44]. The RCTs included in this meta-analysis were designed for the purpose of establishing drug efficacy, and they are thus of short duration; the average length of patient follow-up was 1.62 person-years per subject. MIs are rare enough events that studies with short follow-up time are unlikely to have the power to detect differential risk. Indeed, the meta-analysis itself reports only 62% power to detect an odds ratio of 1.8 for MI [9], [44]. As each individual trial is relatively small, the number of events for many of the trials is 0 for both exposure groups, again reflecting the scarcity of information.

The evidence for risk from cumulative exposure to abacavir is also mixed. Only three studies reported on this relationship, with conflicting point estimates. Lang reported no association with cumulative abacavir use (OR = 0.97, 95% CI: 0.86, 1.1) [8], whereas DAD reported a relative risk of 1.14 per year (95% CI: 1.08, 1.21) [4]. Bedimo et al., who reported a HR, found no association (HR 1.18, 95% CI: 0.92, 1.50) [43]. The heterogeneity between these fair-quality observational studies suggests that there is still uncertainty about the cumulative risk of MI from abacavir. An additional question is how to reconcile uncertainty about cumulative risk with the finding that recent exposure to abacavir is associated with increased risk. A possible explanation might be that those who remain on abacavir have cardiovascular risk profiles that continue to be favorable while on the regimen and that those whose profiles become unfavorable while on abacavir are removed from the regimen; if those with unfavorable profiles were more likely to experience an MI, it would thereby implicate recent use of abacavir but allow cumulative use to not appear harmful. In conclusion, the available evidence on the association between abacavir use and MI is not definitive.

### Exposure to PIs

Some of the studies investigating PIs found an association between cumulative use of PIs and cardiovascular disease, and others found an association with recent exposure. Our meta-analysis based on three observational studies indicated that recent PI use was associated with an odds ratio of 2.13 for MI. We caution, however, that this combined estimate is based upon studies that did not meet important criteria for quality [23]–[25]. In contrast to the findings from observational studies, Coplan et al. conducted a meta-analysis of RCTs [20] and found no association between nelfinavir and risk of MI (point estimate not reported) or between indinavir exposure and risk of MI (0.7, 95% CI: 0.1, 7.75). Similar issues present here as with the meta-analysis of RCTs presented by Ding et al. that evaluated the association between abacavir and MI [9]. These include drawing inference from studies of short duration that are not designed to evaluate endpoints such as MI and that consequently are underpowered to detect such associations.

Finally, we found a significant increase in risk associated with cumulative lopinavir and indinavir use. These results are based upon only two studies, and their quality was fair [6], [8]. We therefore caution against interpreting these findings as conclusive.

### Methodological Challenges

When possible, we combined estimates from studies to assess the risk of cardiovascular disease associated with ART. There were significant challenges, however, to achieving this in our study. Barriers included heterogeneity across the studies with regard to definitions of drug exposure (e.g., time-varying or fixed; cumulative exposure or recent exposure), populations investigated, designs employed (e.g., longitudinal or cross-sectional), and finally, specification of the statistical models (e.g., assessing cumulative or recent exposure separately or jointly). While some of the studies allowed the association of drug exposure to vary over time (e.g., DAD, Lang, Bedimo, and Choi) [4], [8], [30], [43], some considered drug exposure to be a fixed effect (e.g., Daftary and Holmberg) [23], [24]. Incorporating information about how exposure changes over time for an individual (e.g., whether the subject is currently exposed to the drug at a specific time point versus whether the subject was ever exposed over their observation period) will impact the estimates of the coefficients, their interpretation, and therefore their comparability across studies. In addition, some of the studies were based on models that specified risk as a function of cumulative exposure to a drug whereas others were based on models that specified risk as a function of both cumulative exposure as well as indicators for past and recent exposure. Cumulative exposure in the former model represents the change in risk corresponding to a 1-year increase in exposure and includes the risk for someone exposed for 1 year relative to someone never exposed. Cumulative exposure in the latter model, on the other hand, represents the change in risk corresponding to a 1-year increase in exposure only among those exposed.

An illustration of the impact of these analytic choices on findings may be provided by the discrepancy in estimates obtained by Bedimo et al. and Choi et al. [30], [43]. Both studies were conducted using the same data on the same population – the national sample of HIV-infected US veterans (with some differences in inclusion/exclusion criteria as well as follow-up time). Bedimo et al. estimated the effect of recent exposure to abacavir on the hazard of MI relative to exposure to neither abacavir nor tenofovir and demonstrated no association with a point estimate indicating protection among abacavir users against acute MI (HR = 0.67; 95% CI: 0.43, 1.03). In contrast, Choi et al. estimated the effect of recent abacavir exposure on the hazard of MI to be 1.64 (95% CI = 0.88–3.08). It is difficult to say whether these differences can be attributed to how the patients were selected from the larger cohort, the chosen reference group for comparisons, how the models were specified including which covariates were used for adjustment, how exposure was defined, or a combination of all of these factors. However, this discrepancy exemplifies well some of the challenges faced with combining evidence across studies to evaluate the effect of ART on MI.

An additional issue with these analyses involved the potential for confounding by indication. In the observational setting, patients with HIV start and stop regimens and initiate new ones over time. Factors governing such decisions likely relate to a patient’s response to treatment. When decisions for initiation or termination of regimens are related to risk of MI, however, drawing inference about risk of MI may be confounded by one’s history of treatment and risk factor profile. Tools such as marginal structural models and propensity score methods may be useful in addressing this issue [45]–[52]. For example, Obel et al. [21] incorporated the use of propensity scores in their analysis, and we advocate the use of such methods to attempt to handle this complex confounding. Of course, application of these methods designed to handle challenging statistical issues is no guarantee of valid findings and also rely on their own sets of assumptions. We therefore recommend sensitivity analyses that vary methods, which rely on different assumptions, and that vary definitions of drug exposure. This can provide insight into the robustness of findings to different assumptions. Some of the studies described here made use of such approaches to provide insight into the interpretation of their results [4], [8].

Finally, we should note that there is some overlap between our study and that by Islam et al. who address topics similar to those discussed in our paper [53]. While our work highlights comparisons of the risks of MI among HIV-positive people exposed to different regimens of ART, the study by Islam et al. has a different scope that also includes comparisons between HIV-positive and HIV-negative subjects. Where our goals overlap, however, our findings are largely in agreement. Islam et al. observed an increased risk of CV events with exposure to PIs as a class, lopinavir, and abacavir, and our analysis additionally revealed an increased risk in MI from exposure to indinavir. However, our findings are based on methodological choices regarding pooling or summarizing estimates that differ from those of Islam and colleagues. We chose not to combine results from studies that reported hazard ratios with studies that reported odds ratios or relative risks, due to concerns about the interpretability of a summary statistic. We also chose not to use a study more than once in any specific summary estimate, even if it had relevant data (for example, two NRTI drugs), because of concerns that the lack of independence of observations could lead to underestimation of the standard errors for the summary ORs. In sum, although we made different methodological choices, our findings are largely in agreement with those of Islam and colleagues.

### Conclusion

Our study found evidence based on observational studies to suggest a harmful association between abacavir and risk of MI. In addition, there is evidence from observational studies that use of PIs increases risk of MI. Evidence from the observational and randomized trials are at odds, however. While the randomized clinical trial setting would provide the least biased approach to assessing cardiovascular risk, the clinical trials included in our investigation were not designed for that purpose. Consequently they were short-term, and were underpowered for assessing cardiovascular risk. Compared to these meta-analyses of RCTs, the observational studies include much longer follow up and a more representative sample, but they are subject to confounding by indication, in which risk of cardiovascular disease may influence both ART choice and MI, which may lead to spurious associations. In addition, combining evidence across studies proves challenging in the presence of heterogeneity of study designs and analytic plans. Based on the overall evidence, we believe there is still uncertainty whether ART leads to increased cardiovascular risk, and if so, the magnitude of that risk. The current evidence provided by the observational studies is sufficient, however, to warrant further study in prospective studies designed to assess cardiovascular risk from ART.

## Supporting Information

Supporting Information (DOC)
